# Supplementary material for: Physical Functional Ability and Quantitative Assessment of the Multifidus Muscle of the Lumbar Spine in the Elderly
Source: Diagnostics (Basel). 2023 Jul 20;13(14):2423. doi: 10.3390/diagnostics13142423 (PMC10378454; doi:10.3390/diagnostics13142423)
Supplement: Supplementary file 1 [file diagnostics-13-02423-s001.zip › diagnostics-2493779-supplementary.pdf]

Table S1. CSA and fCSA of multifidus muscle and the CSA to fCSA ratio, classified according to Pfirrmann Classification of lumbar disc of each level.

	L2-3			L3-4			L4-5			L5-S1		
Pfirrmann Classification	n	Mean±SD	F(p)	n	Mean±SD	F(p)	n	Mean±SD	F(p)	n	Mean±SD	F(p)
Multifidus												
2	7	621.21±177.42	0.964	3	1097.98±201.58	1.259	4	1481.78±302.65	0.701	2	1796.25±411.56	0.488
3	22	726.16±179.85	(0.434)	24	1142.14±385.91	(0.361)	22	1545.01±392.37	(0.569)	18	1577.78±569.22	(0.705)
4	22	757.15±193.26		27	978.26±217.83		23	1381.80±363.43		24	1582.67±430.60	
5	6	753.35± 173.79		3	1076.77±97.57		8	1408.06 ±273.70		13	1417.45±485.61	
Functional Multifidus												
2	7	456.25±180.54	0.710	3	824.28±251.51	1.952	4	1025.07±365.30	0.949	2	1508.42±357.14	1.454
3	22	563.04±158.67	(0.560)	24	920.74±382.58	(0.230)	22	1132.76±407.64	(0.449)	18	1115.24±514.52	(0.333)
4	22	546.52±204.59		27	691.43±224.42		23	942.56±318.01		24	1113.47±382.73	
5	6	586.77±170.92		3	664.90±227.83		8	1058.40±309.65		13	902.68±414.10	
Ratio												
2	7	71.48±11.24	1.706	3	74.02±12.19	4.035	4	68.14±14.64	0.758	2	83.90±0.66	32.120
3	22	77.50±10.56	(0.361)	24	79.63±9.32	(0.081)	22	72.14±10.44	(0.540)	18	68.91±11.12	**(<0.001)**
4	22	70.70±11.16		27	69.57±10.48		23	68.16±12.46		24	69.78±10.12	
5	6	77.26±7.47		3	60.83±16.77		8	74.18±9.31		13	62.21±14.01	

Ratio (%): CSA of multifidus muscle/CSA of functional multifidus muscle x100. Abbreviations: CSA, cross-sectional area
